# Characterization of Two TNF-Related Subtypes Predicting Infliximab Therapy Responses in Crohn’s Disease

**DOI:** 10.3389/fimmu.2022.871312

**Published:** 2022-04-22

**Authors:** Chenglin Ye, Sizhe Zhu, Jingping Yuan

**Affiliations:** ^1^ Department of Pathology, Renmin Hospital of Wuhan University, Wuhan, China; ^2^ Department of Clinical Immunology, Tongji Hospital, Tongji Medical College, Huazhong University of Sciences and Technology, Wuhan, China

**Keywords:** TNF family, Crohn’s disease, anti-TNF therapy, subtypes, immune infiltration

## Abstract

**Background:**

Anti–tumor necrosis factor (TNF) therapy is widely used to treat Crohn’s disease (CD). Unfortunately, 10%–40% of patients have primary non-response to anti-TNF therapy. TNF family genes play crucial roles in inflammation and immune regulation; however, the effects of TNF family genes on CD remain unclear.

**Methods:**

CD expression profiles were downloaded from the Gene Expression Omnibus database. Unsupervised clustering was then used to identify the gene subtypes in CD based on the expressions of TNF family genes. The features of the gene subtypes were characterized using functional enrichment and immune infiltration analyses, and biomarkers of the gene subtypes were identified.

**Results:**

Patients with CD were divided on the basis of unsupervised clustering into two gene subtypes: immune and metabolic. Gene subtype A was significantly correlated with leukocyte migration and cytokine interactions, whereas gene subtype B was associated with metabolic pathways. Whereas 89.5% of the patients in gene subtype B responded to infliximab, only 16.7% of patients in gene subtype A responded. In addition, a combination of interleukin 1 beta (IL1β), interleukin 6 (IL6), and Toll-like receptor 4 (TLR4) can effectively distinguish between gene subtypes A and B.

**Conclusion:**

Comprehensive analyses of the TNF family genes may reveal the underlying pathogenesis of CD. The classification of subtypes may provide new ideas for the personalized treatment of patients with CD.

## Introduction

Crohn’s disease (CD), a chronic gastrointestinal inflammatory disease, is a subtype of inflammatory bowel disease (IBD). It is characterized by a recurrent cycle of uncontrolled inflammation (1). The incidence of CD has increased in several parts of the world. The current estimated incidence rate is 0.51–1.09 per 100,000 population in China (2, 3). Until now, the medical treatment for CD usually has included aminosalicylates, antibiotics, corticosteroids, immunomodulators, and biologic agents. Anti–tumor necrosis factor (TNF) inhibitors as biologic agents are effective for treatment of patients with CD who respond inadequately to treatment with corticosteroids and immunomodulators (4). Infliximab, a TNF inhibitor, was approved for CD treatment in 1998 and improved response and remission rates in patients (5). Unfortunately, 10%–40% of patients have primary non-response to anti-TNF therapy. In addition, almost half of the patients who respond to anti-TNF therapy will lose clinical benefits within the first year (6, 7). The reasons for treatment failure are still a matter of uncertainty.

The TNF family comprises the TNF ligand superfamily (TNFSF) and TNF receptor superfamily (TNFRSF), which include 19 ligands and 29 receptors, respectively (8). The TNF family plays important roles in modulating cellular functions and is involved in inflammation and cancer pathogenesis (9). TNF expression is upregulated in both the colon mucosa and blood serum of patients with CD. In addition, TNF contributes to disruption of the intestinal epithelial barrier and is a major effector molecule involved in the pathogenesis of CD (10). In colitis, lack of the TNF receptor, TNF receptor II (TNFRII), in T cells worsens the disease (11). Furthermore, a significant effect of TNFRSF1A/1B on the response to anti-TNF therapy in patients with CD has been reported (12). However, the functions of the TNF family in CD and their effect on anti-TNF therapy response remain to be explored.

In this study, we aimed to comprehensively evaluate the function of TNF family members in CD. Two TNF subtypes were identified on the basis of the expression of the 43 TNF family genes. Furthermore, we classified patients with CD into two gene subtypes based on the differentially expressed genes (DEGs) of the two TNF subtypes. We further analyzed the percentage of patients who responded to infliximab therapy in the two gene subtypes. Moreover, biomarkers with excellent discrimination between the two gene subtypes were identified.

## Method

### Data Collection

The gene expression datasets of CD were downloaded from the Gene Expression Omnibus database using the search terms “CD and anti-TNF” or “IBD”. The obtained datasets included the following: GSE36807 (13 CD tissues and seven normal tissues) (13), GSE98820 (31 CD inflamed tissues before and after adalimumab treatment), GSE102133 (65 CD tissues and 12 normal tissues) (14), GSE186582 (464 CD tissues that were treated or untreated by anti-TNF therapy and 25 normal tissues), GSE16879 (213 CD inflamed tissues before and after infliximab treatment and 13 normal tissues) (15), and GSE111761 (lamina propria mononuclear cells isolated from six CD tissues) (16). The GSE16879 and GSE111761 datasets contained clinical information about whether patients responded to infliximab treatment. The patients were classified according to their response to infliximab. This classification was based on endoscopic and histologic findings 4–6 weeks after first infliximab treatment. The patients who respond to infliximab was defined as a complete mucosal healing with a decrease of at least three points on the histological score, and the patients who did not achieve this healing were considered non-responders. The ComBat function of the sva R package was used to merge the GSE36807, GSE98820, GSE102133, and GSE186582 datasets and remove batch effects (17).

### Gene Set Variation Analysis and Single-Sample Gene Set Enrichment Analysis

Pathway enrichment in each sample was revealed using gene set variation analysis (GSVA) (18). Single-sample gene set enrichment analysis (ssGSEA) was used to estimate immune cell infiltration; the gene signatures of immune cells are listed in **Supplementary Table S1** (19).

### Unsupervised Clustering for TNF 7Family Genes

Unsupervised clustering, which included 43 TNF family genes, was conducted to identify different TNF subtypes. A consensus clustering algorithm was performed using the R package ConsensusClusterPlus (20) and was repeated 1,000 times to ensure the clustering stability. The group information after unsupervised clustering of the datasets is shown in **Supplementary Table S2**.

### Differential Analysis and Weighted Correlation Network Analysis

Differential analyses were performed using the Limma R package (21). Genes with an adjusted p-value <0.05 and | log_2_(fold change)| >0.5 were defined as DEGs. Weighted correlation network analysis (WGCNA) was used to identify related modules (22); the minimum number of module genes was set at 30. The hierarchical clustering dendrogram summarizes the gene modules with different colors.

### Functional Enrichment Analysis and Protein–Protein Interaction

Gene ontology (GO) and Kyoto Encyclopedia of Genes and Genomes (KEGG) pathway enrichment analyses were performed using the clusterProfiler and GOplot R packages (23, 24). GSEA was conducted using the clusterProfiler R package. Enrichment datasets were obtained from MSigDB. A protein–protein interaction (PPI) network was constructed using the STRING database (25) and visualized using Cytoscape 3.8.2. A Cytoscape plug-in, Molecular Complex Detection (MCODE), was also used to identify the hub clusters of the PPI network.

## Results

### Unsupervised Clustering for TNF Family Genes and Immune Infiltration Analysis

**Figure 1:**
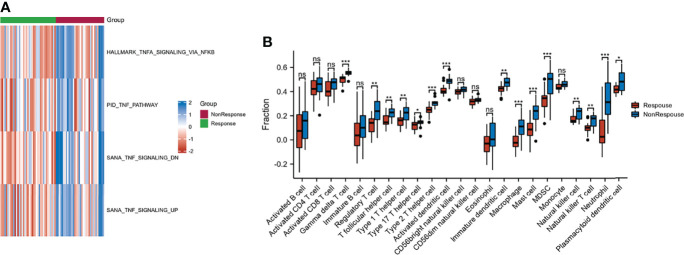
Characteristic of response and non-response to anti-TNF therapy in patients with CD. **(A)** GSVA of TNF-related pathway between response and non-response groups; blue and red represent activated and inhibited pathways, respectively. **(B)** Infiltration fraction of immune cell in response and non-response patients (*p < 0.05, **p < 0.01, and ***p < 0.001; ns, not significant).

**Figure 2:**
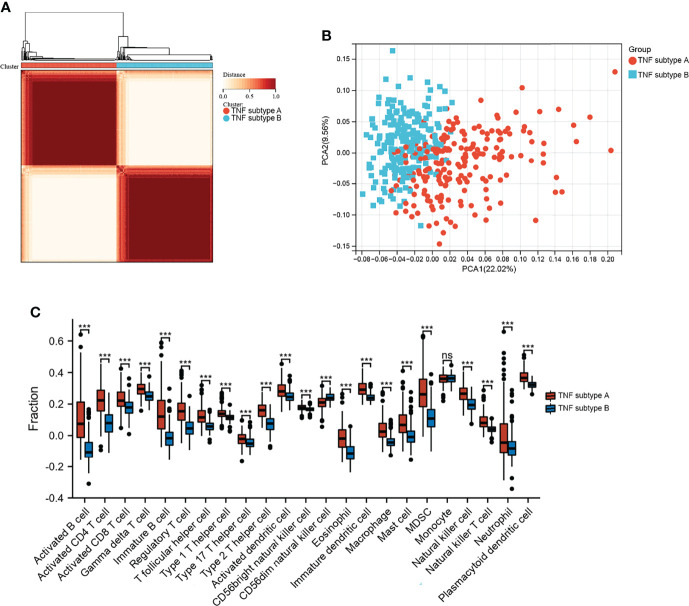
Identification of TNF subtypes in CD. **(A)** Consensus clustering matrices of 43 TNF family genes in CD merged dataset (k = 2). **(B)** PCA for the expression of 43 TNF family genes to distinguish two TNF subtypes in CD merged dataset. **(C)** Infiltration fraction of Immune cells in two TNF subtypes (***p < 0.001; ns, not significant).

GSVA was performed to explore the functions of TNF family genes in CD samples from patients who did or did not respond to infliximab therapy in GSE16879. **Figure 1A** shows that the GSVA scores of TNF-related pathways in the non-response group were significantly increased compared to the response group. The infiltration of immune cells in the two groups is presented in **Figure 1B** and **Supplementary Table S3**. The non-response group showed higher immune cell infiltration. Furthermore, to identify novel subtypes, 412 patients with CD without anti-TNF treatment from the merged datasets (GSE36807, GSE98820, GSE102133, and GSE186582) were integrated. The unsupervised clustering method was applied on the basis of the expression files of the 43 TNF family genes. **Figure 2A** and **Supplementary Figure S1** show that the two TNF subtypes achieved the best clustering efficacy in merged datasets. Principal component analysis (PCA) revealed significant differences in the transcription profiles of the TNF family genes between the two TNF subtypes (**Figure 2B**). ssGSEA was performed to explore the differential immune infiltration between the two TNF subtypes. **Figure 2C** shows that 22 of the 23 common immune cells were significantly different between the TNF subtypes; TNF subtype A had a relatively higher infiltration level. The infiltration of immune cells in the merged dataset is listed in **Supplementary Table S4**. Interestingly, no differences in the mRNA expression of CD-associated cytokines were observed between the two TNF subtypes, IL1β, IL6, TNF, C-X-C Motif Chemokine Ligand 1(CXCL1), C-C Motif Chemokine Ligand 5 (CCL5), and C-X-C Motif Chemokine Ligand 16 (CXCL16) (**Supplementary Figure S2A**).

### Identification of Response-Related DEGs

**Figure 3:**
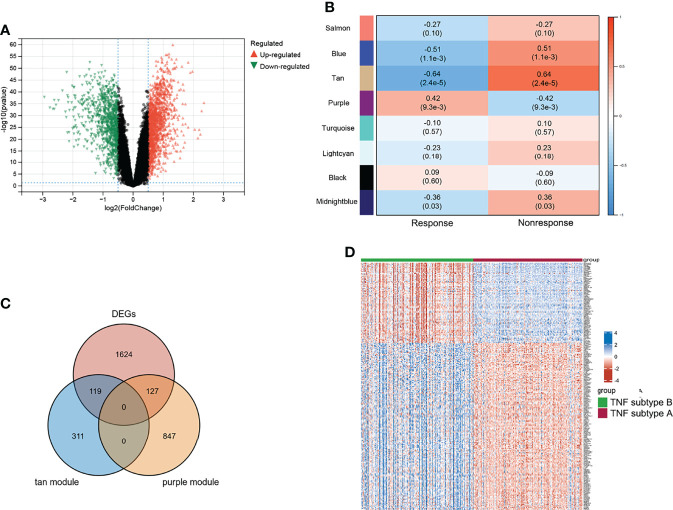
Characteristic of TNF subtypes. **(A)** Volcano plot of DEGs between two TNF subtypes. **(B)** WGCNA module trait relationships in response and non-response, which contained the corresponding correlation and p-value. **(C)** Intersection of DEGs and response-related genes. **(D)** Heatmap of response-related DEGs; blue and red represent upregulated and downregulated expression, respectively.

Differential analyses were performed using the limma R package. **Figure 3A** shows 1,083 upregulated and 787 downregulated DEGs that were identified. We conducted WGCNA based on the expression profile of GSE16879 to screen for genes correlated with infliximab therapy response in patients with CD. The soft threshold β was set as 2 to construct a scale-free network, whereas no scale R^2^ = 0.88 (**Supplementary Figure S2B**). The resulting gene dendrograms and module colors are presented in **Supplementary Figure S2C**, and eight gene modules were identified. **Figure 3B** shows the purple module was positively correlated with infliximab therapy response (module trait correlation = 0.42), whereas the tan module exhibited a strong negative correlation (module trait correlation = −0.64). The intersection of DEGs with the purple and tan modules was defined as response-related DEGs for further analyses (**Figure 3C**). A heatmap of the response-related DEGs is shown in **Figure 3D**.

### Functional Enrichment Analysis and PPI Construction

**Figure 4:**
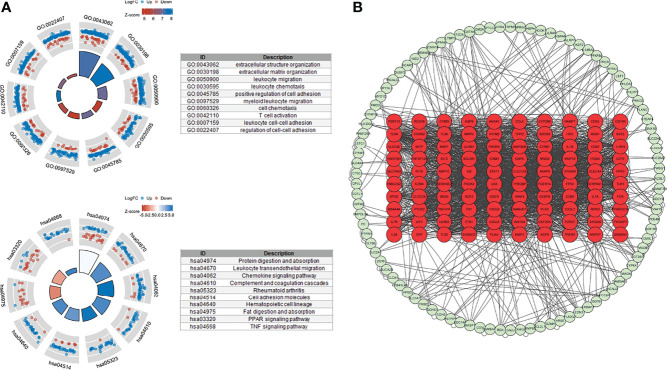
Functional enrichment and PPI networks. **(A)** Top 10 GO BP and KEGG enrichments. **(B)** PPI network of response-related DEGs; top 100 nodes were presented by red circles.

We used 246 representative DEGs for GO and KEGG enrichment analyses to explore the potential biological processes (BPs) of the two TNF subtypes. As shown in **Figure 4A**, representative DEGs were significantly enriched in immune-related BP. KEGG analysis indicated enrichment of immune-, digestion-, and absorption-related pathways (**Figure 4A**). The PPI network constructed using the STRING database is shown in **Figure 4B**. We analyzed the degrees of nodes in the PPI network, and the top 100 nodes are represented by red circles and listed in **Supplementary Table S5**.

### Identification of Gene Subtypes and Immune Infiltration Analysis

**Figure 5:**
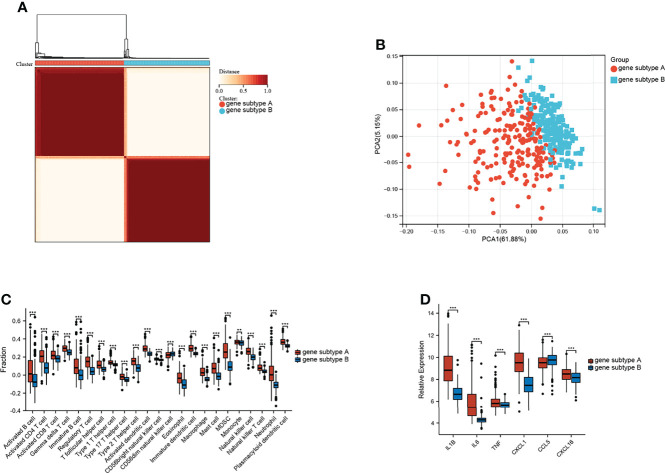
Identification of gene subtypes. **(A)** Consensus clustering matrices of top 100 nodes in CD merged dataset (k = 2). **(B)** PCA for the expression of top 100 nodes to distinguish two genes subtypes in CD merged dataset. **(C)** Infiltration fraction of immune cell in two genes subtypes. **(D)** mRNA expressions of CD-associated inflammatory factor (**p < 0.01 and ***p < 0.001).

Unsupervised clustering analysis was performed on the basis of the expression of the top 100 representative DEGs, which were defined as gene subtypes, to explore the heterogeneity of the different gene subtypes further. **Figure 5A** and **Supplementary Figure S3** show that these two gene subtypes achieved the best clustering efficacy in the merged datasets. PCA analysis revealed significant differences between the two gene subtypes (**Figure 5B**). The correlations between the two gene subtypes and 22 common immune cells in each CD sample were analyzed using ssGSEA. **Figure 5C** shows that gene subtype A had higher infiltration levels of 22 immune cells; only infiltration levels of CD56 dim natural killer cells were lower than those of gene subtype B. Moreover, the mRNA expressions of CD-associated inflammatory factors, such as IL1β, IL6, TNF, CXCL1, and CXCL16, were significantly upregulated, whereas CCL5 was downregulated in gene subtype A (**Figure 5D**).

### Functional Enrichment Analysis and PPI Construction

**Figure 6:**
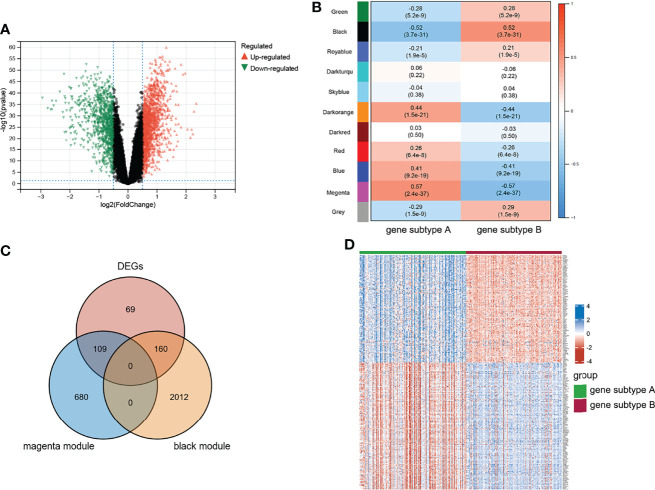
Characteristic of genes subtypes. **(A)** Volcano plot of DEGs between two genes subtypes. **(B)** WGCNA module trait relationships in gene subtype A and B, which contained the corresponding correlation and p-value. **(C)** Intersection of DEGs and subtypes related genes. **(D)** Heatmap of representative DEGs; blue and red represent upregulated and downregulated expression, respectively.

**Figure 7:**
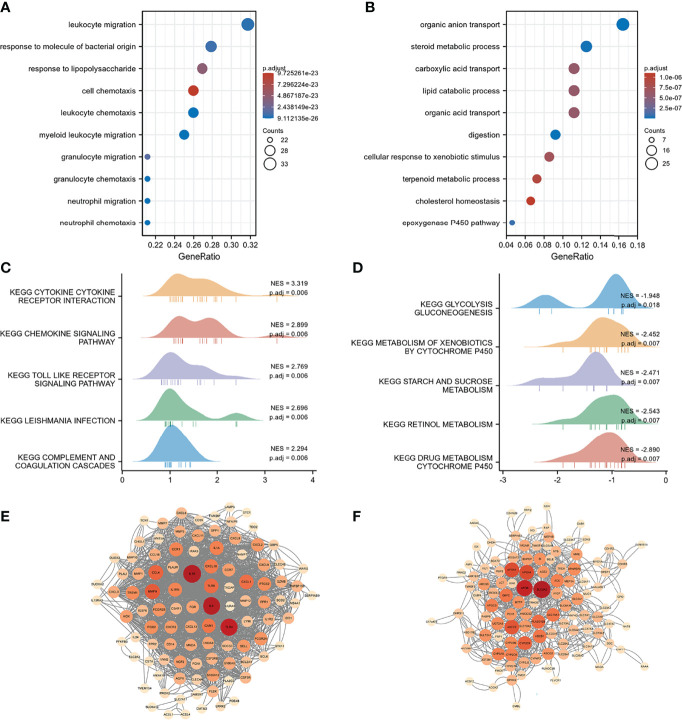
Functional enrichment and PPI networks. **(A)** Top 10 GO BP of gene subtype A. **(B)** Top 10 GO BP of gene subtype B. **(C)** Top 10 GSEA enrichments of gene subtype A. **(D)** Top 10 GSEA enrichments of gene subtype B. **(E)** PPI networks of representative DEGs of gene subtype A. **(F)** PPI networks of representative DEGs of gene subtype B. Light color represents low-degree mapping, whereas dark color represents high-degree mapping.

**Figure 8:**
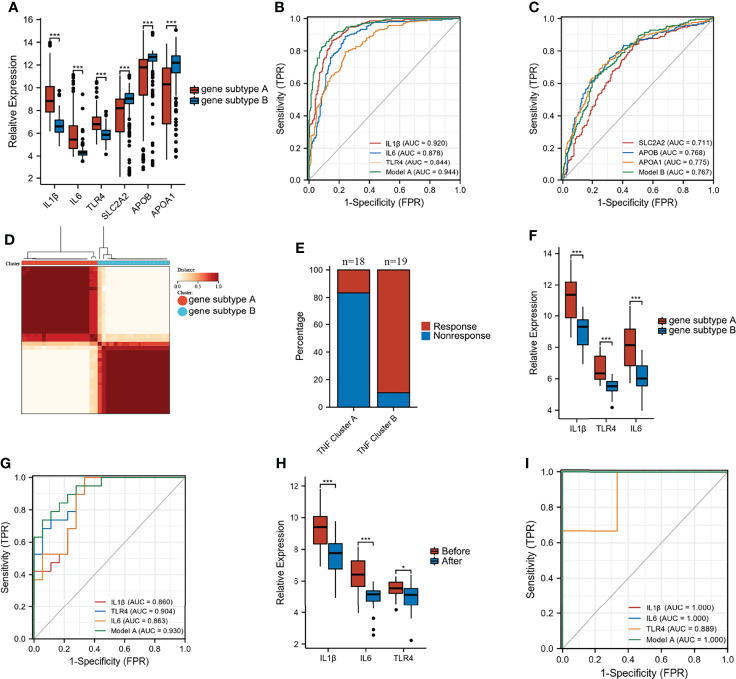
Predictive and classify values of biomarkers. **(A)** The expression of six biomarkers in gene subtype A and B. **(B, C)** ROC curves to classify gene subtypes in merged dataset. **(D)** Consensus clustering matrices of top 100 nodes in GSE16879 (k = 2). **(E)** Stacked bar plot of percentage of response and non-response patients in two gene subtypes. **(F)** The expression of IL1β, IL6, and TLR4 in two gene subtypes in GSE16879. **(G)** ROC curves to classify gene subtypes in GSE16879. **(H)** The expression of IL1β, IL6, and TLR4 in response group before and after treatment. **(I)** ROC curves to classify gene subtypes in validation dataset GSE111761 (*p < 0.05 and ***p < 0.001).

Differential analysis and WGCNA were used to identify the representative DEGs of these two gene subtypes to explore further the two gene subtype characteristics (**Figures 6A, B**). A total of 109 and 160 representative DEGs were identified for gene subtypes A and B, respectively (**Figures 6C, D** and **Supplementary Table S6**). These representative DEGs were further used to conduct GO enrichment analysis and GSEA; gene subtype A was significantly enriched in BP correlated with immunity, whereas gene subtype B had metabolism-related pathway enrichment (**Figures 7A–D**). The PPI networks of gene subtypes A and B are presented in **Figures 7E, F**, respectively. The top three genes [interleukin 1 beta (IL1β), interleukin 6 (IL6), and Toll-like receptor 4 (TLR4)] ranked by degree were selected as feature genes for gene subtype A, whereas SLC2A2, APOB, and APOA1 were feature genes of gene subtype B. Moreover, the expressions of IL1β, IL6, and TLR4 were significantly upregulated, and SLC2A2, APOB, and APOA1 were downregulated in gene subtype A (**Figure 8A**). We further explored the ability of these six genes to distinguish between gene subtypes A and B. **Figures 8B, C** show that the combination of IL1β, IL6, and TLR4 (AUC of Model A = 0.944) had a better ability to distinguish between gene subtypes A and B than the combination of SLC2A2, APOB, and APOA1 (AUC of Model B = 0.767). Therefore, IL1β, IL6, and TLR4 were identified as biomarkers for distinguishing between gene subtypes A and B.

### Infliximab Therapy Response Analysis

Unsupervised clustering analysis was performed based on the top 100 nodes in GSE16879 to explore the differential response to infliximab therapy between gene subtypes A and B. **Figure 8D** shows that these two clusters achieved the best clustering efficacy in GSE16879. The group information after unsupervised clustering of the GSE16879 dataset is presented in **Supplementary Table S7**. We investigated the number of patients who did or did not respond to infliximab therapy in gene subtypes A and B. Of the patients, 16.7% (n = 18) and 89.5% (n = 19) responded to infliximab therapy in gene subtypes A and B, respectively (**Figure 8E**). The expression levels of IL1β, IL6, and TLR4 are shown in **Figure 8F**. The AUC of Model A (combination of IL1β, IL6, and TLR4) was 0.930 in GSE16879 (**Figure 8G**). Moreover, the relative expressions of IL1β, IL6, and TLR4 were significantly downregulated after infliximab therapy in the response group, and there was no difference in the non-response group (**Figure 8H** and **Supplementary Figure S5A**). Heatmaps of the top 100 genes in the response and non-response groups before and after treatment are presented in **Supplementary Figures S5B, C**. We further validated that GSE 111761, IL1β, IL6, and TLR4 could easily distinguish responders and non-responders (**Figure 8I**). The expression of IL1β, IL6, and TLR4 in the response and non-response groups is presented in **Supplementary Figure S5D**. Importantly, this study demonstrated that patients with gene subtype B had superior infliximab therapy response.

## Discussion

CD is a chronic gastrointestinal inflammatory disease that has no effective treatment. Anti-TNF therapy is the preferred treatment option for patients with CD who present with moderate-to-severe disease activity and are intolerant of, or do not respond to, corticosteroids or immunosuppressants, or are steroid-dependent. Although anti-TNF therapy improves clinical success, healing rates of the mucosa, and quality of life in patients with CD, 10%–40% of patients primarily do not respond to anti-TNF therapy (26). Therefore, novel models and biomarkers for predicting the effects of anti-TNF treatment are urgently required.

TNF family genes and TNF-related signaling pathways play a vital role in inflammation and immune regulation (8). TNF is the main cytokine in the pathogenesis of CD (27). TNF-related apoptosis-inducing ligand (TRAIL) has also been designated as TNFSF10, possibly contributing to the development of fistulas and strictures in patients with CD by disrupting intestinal epithelium integrity and inducing epithelial cell apoptosis (28). In a 2,4,6-trinitrobenzenesulfonic acid (TNBS)–induced colitis mouse model, the lack or blocking of TNF-related weak inducer of apoptosis (Tweak), also known as TNFSF12, resulted in the expression of pro-inflammatory cytokines and a decrease in inflammatory cell infiltration in the intestine (29). However, most studies have focused on a single TNF family ligand and receptor. The overall effect of multiple TNF family genes on CD has not been fully elucidated. We first analyzed the enrichment scores of TNF-related pathways in patients with CD who did or did not respond to infliximab therapy. The results demonstrated that TNF-related pathways were significantly activated in the non-response group. Considering the significant difference, we further identified two TNF subtypes based on the expression of 43 TNF family genes in CD. Immune infiltration was obviously different between the two TNF subtypes. However, CD-associated cytokines between two TNF subtypes, such as IL1β, IL6, TNF, CXCL1, CCL5, and CXCL16, were not differentially expressed.

To further explore the heterogeneity of CD, infliximab therapy response-related DEGs that were significantly enriched in immune-related BP were identified. The top 100 DEGs in terms of degrees were used for unsupervised clustering analysis, which divided patients with CD into two groups named gene subtypes A and B. Gene subtype A showed a higher infiltration level of immune cells, similar to the non-response group. This demonstrated that immune cell infiltration was involved in resistance to infliximab therapy. Schmitt et al. found that the expansion of TNFR2^+^IL23R^+^ T cells, which were activated from CD14^+^ macrophages, was involved in resistance to anti-TNF therapy in patients with CD (16). Moreover, Martin et al. constructed a cell module consisting of IgG plasma cells, inflammatory mononuclear phagocytes, activated T cells, and stromal cells. Non-responsive and responsive patients with CD were found to have different cell module scores (30). Non-response of patients with CD to anti-TNF therapy was reported to be due to an innate transcriptional dysregulation of monocytes, resulting in excessive activation of pro-inflammatory pathways (31). In addition, the expressions of IL1β, IL6, TNF, CXCL1, CCL5, and CXCL16 were significantly different; gene subtype A had a higher level of inflammation than gene subtype B. Lacruz-Guzmán et al. reported that higher concentrations of serum IL1β had a lower response to infliximab treatment in patients with CD (32). Patients with CD in whom anti-TNF therapy failed showed increased expression of IL-6 and persistent IL-6 pathway activity (33). These results indicated that patients with CD with gene subtype B, which had lower immune infiltration levels and inflammation, were more likely to benefit from anti-TNF therapy.

Furthermore, representative DEGs of gene subtypes A and B were identified to characterize the two gene subtypes. Representative DEGs of gene subtype A were significantly enriched in immune-related BP. In contrast, representative DEGs of gene subtype B were enriched in metabolic processes, which was similar to the results of GSEA. This demonstrated that dysregulation of metabolism and immunity may represent defining features of CD subtypes and reflect the heterogeneity of CD, which is consistent with previous findings (34). Moreover, biomarkers of gene subtype A and B were screened, respectively. Among the six biomarkers, IL1β, IL6, and TLR4 had excellent abilities to distinguish gene subtypes A and B. Finally, we used the same method for clustering responsive and non-responsive patients in GSE16879. We noticed that 89.5% of patients in the gene subtype B responded to infliximab therapy. However, only 16.7% of the patients in gene subtype A responded to infliximab therapy. This suggested that patients in gene subtype B may be inclined to respond to infliximab therapy. This was an important finding in our study for classifying and predicting the response to infliximab therapy in patients with CD before treatment.

Nevertheless, this study has several limitations. First, this study was conducted on the basis of the data from a public database; therefore, prospective samples are required for further validation. Second, the dataset for infliximab therapy response analysis had a relatively small sample size. It lacked more data of patients with CD who were responsive/non-responsive to anti-TNF therapy for validation. Finally, data on some important clinical variables, such as disease phenotype, activity and duration, previous bowel resection, smoking, and previous therapies, were unavailable for analysis, which may have affected the prediction of infliximab therapy response.

## Conclusion

Our comprehensive analyses of TNF family genes in CD revealed the underlying regulatory mechanism of the effect on immune infiltration and response to infliximab therapy. The identified gene subtypes and biomarkers showed promising potential for distinguishing and predicting patients who underwent infliximab therapy. These results revealed the clinical implications of the TNF family genes in CD. Importantly, this finding will provide new auxiliary diagnostic indicators and contribute to a novel approach for personalized treatment of CD.

## Data Availability Statement

The datasets presented in this study can be found in online repositories. The names of the repository/repositories and accession number(s) can be found in the article/**Supplementary Material**.

## Author Contributions

CY, SZ, and JY conceived and designed the project. CY and SZ analyzed and interpreted the data. CY and JY wrote and revised the manuscript. All authors contributed to the article and approved the submitted version.

## Conflict of Interest

The authors declare that the research was conducted in the absence of any commercial or financial relationships that could be construed as a potential conflict of interest.

## Publisher’s Note

All claims expressed in this article are solely those of the authors and do not necessarily represent those of their affiliated organizations, or those of the publisher, the editors and the reviewers. Any product that may be evaluated in this article, or claim that may be made by its manufacturer, is not guaranteed or endorsed by the publisher.
