# Supplementary material for: Characterization of Two TNF-Related Subtypes Predicting Infliximab Therapy Responses in Crohn’s Disease
Source: Front Immunol. 2022 Apr 22;13:871312. doi: 10.3389/fimmu.2022.871312 (PMC9072632; doi:10.3389/fimmu.2022.871312)
Supplement: Supplementary file 1 [file DataSheet_1.docx]

Supplementary Material


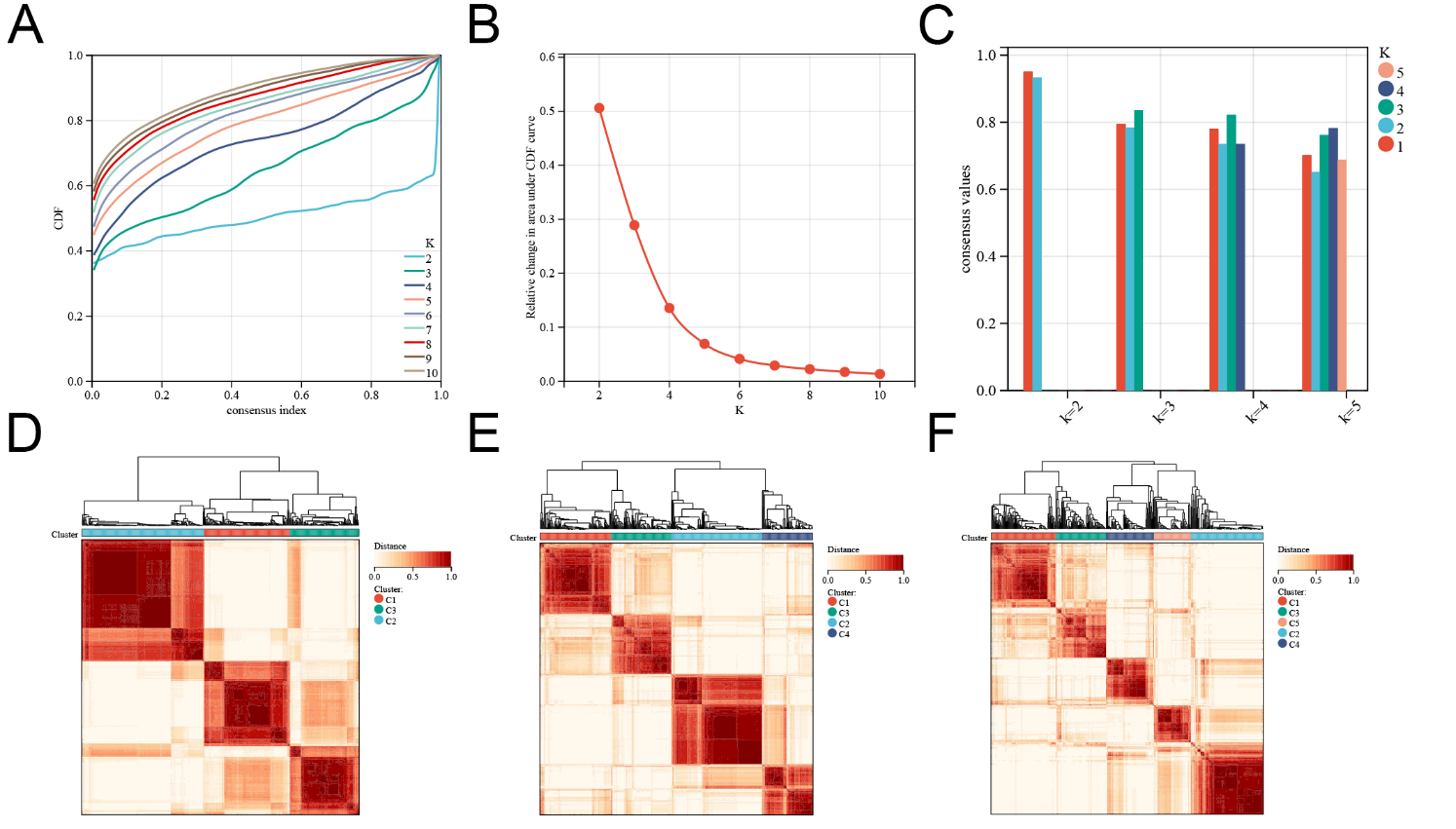


**Supplementary Figure S1.** (A) Cumulative distribution function (CDF) curves. (B) The area under CDF curves (C) The bar plots represent the consensus scores for subtypes with k=2-5. (D-F) Consensus clustering matrices of 43 TNF family genes in CD merged datasets (k=2-5).


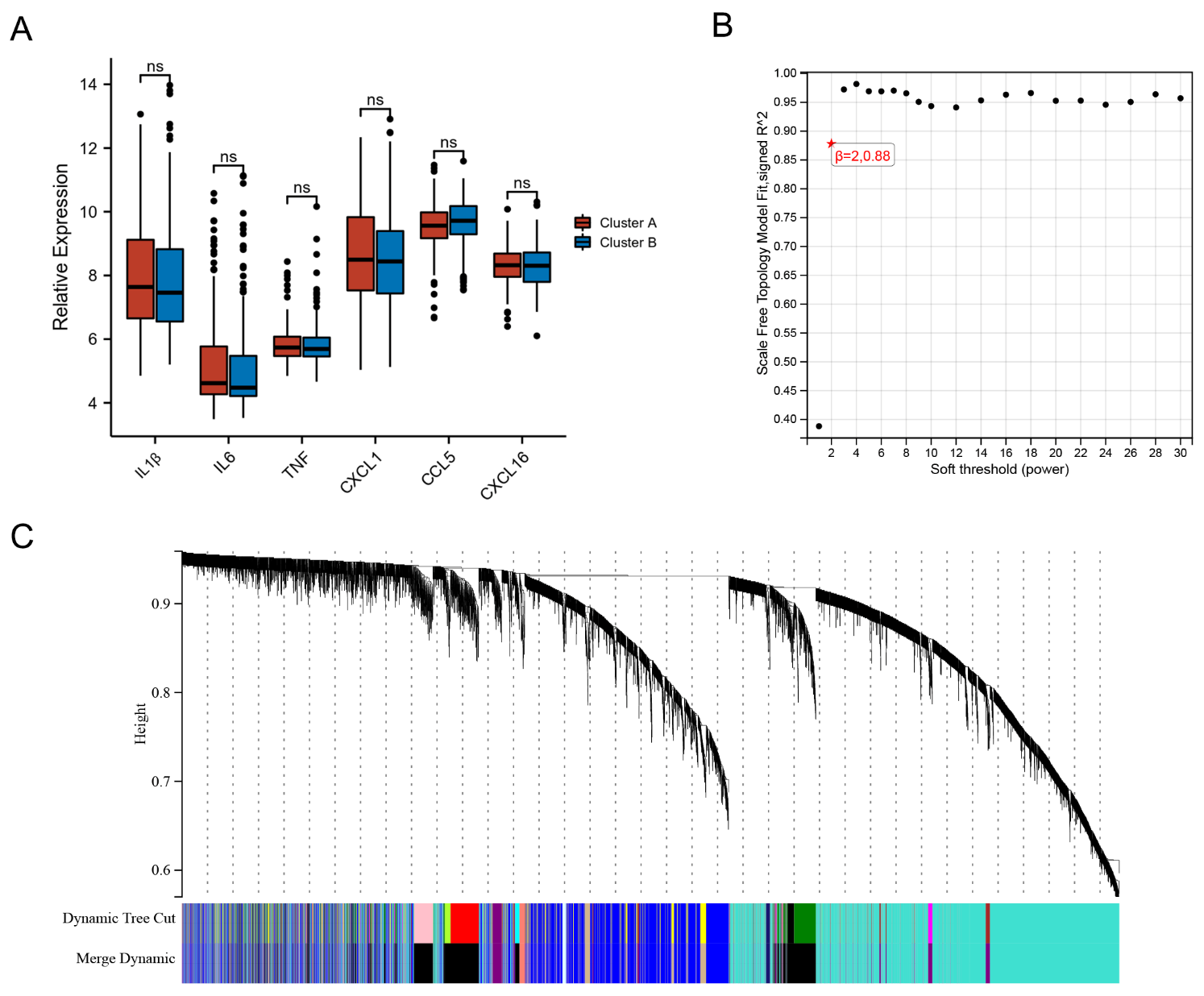


**Supplementary Figure S2.** (A) The relative expression of CD-associated inflammatory factor. (B)WGCNA soft threshold β setting. (C) Resulting gene dendrograms. (ns, not significant)


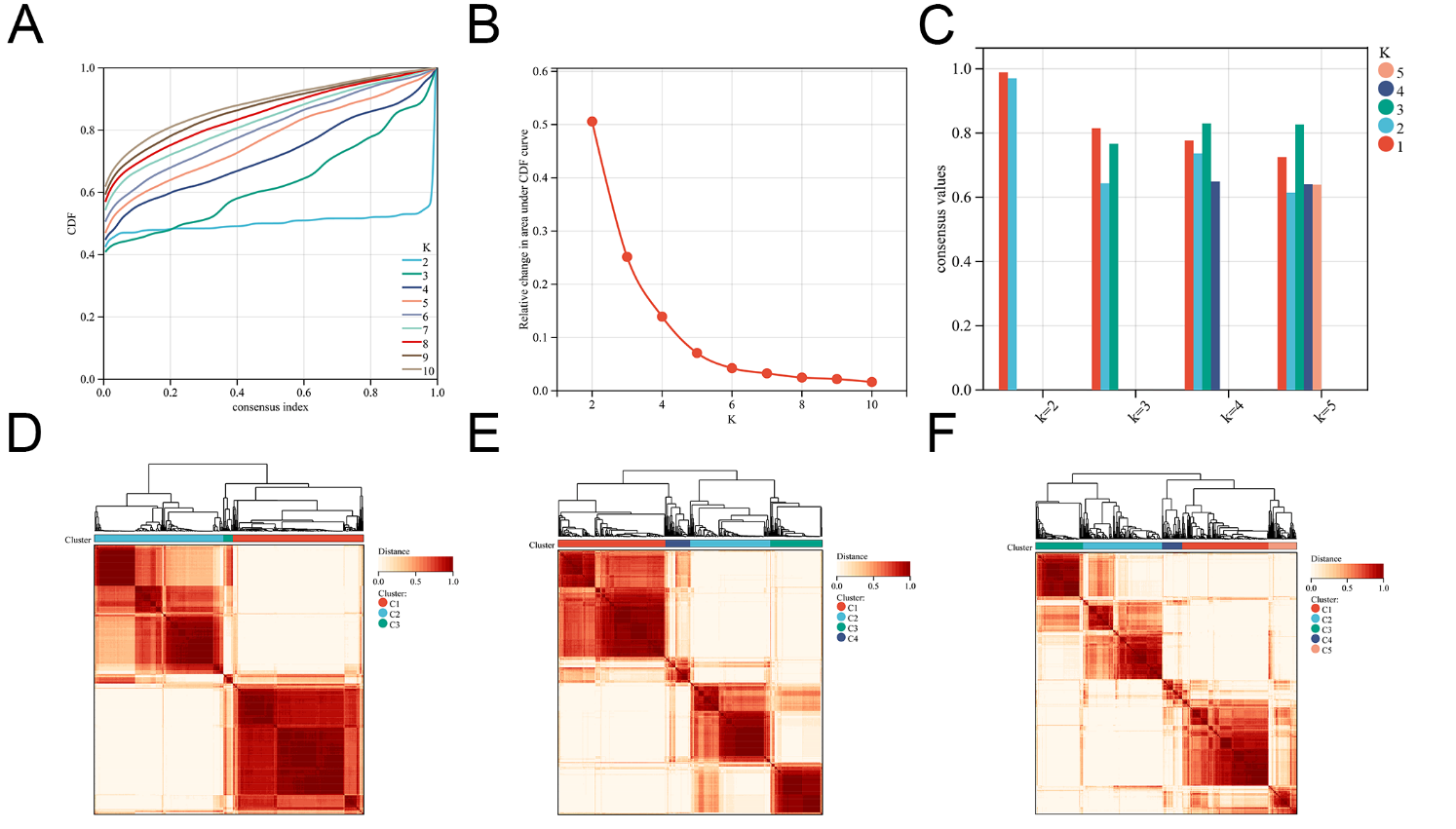


**Supplementary Figure S3.** (A) CDF curves. (B) The area under CDF curves (C) The bar plots represent the consensus scores for subtypes with k=2-5. (D-F) Consensus clustering matrices of top 100 nodes in CD merged datasets (k=2-5).


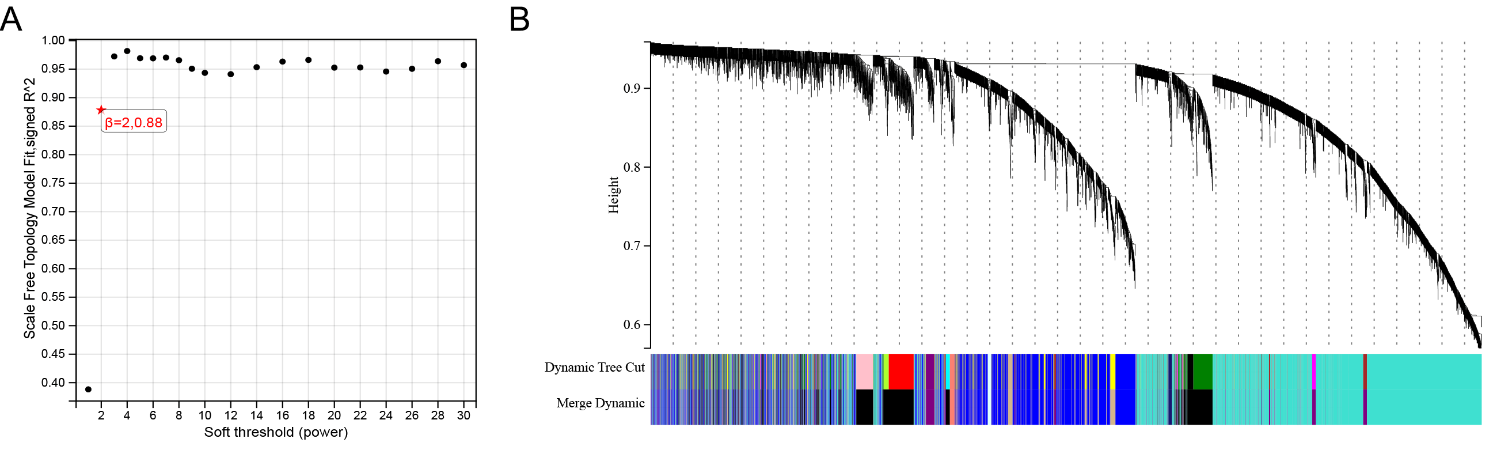


**Supplementary Figure S4.** WCGNA (A) soft threshold β setting. (C) Resulting gene dendrograms.


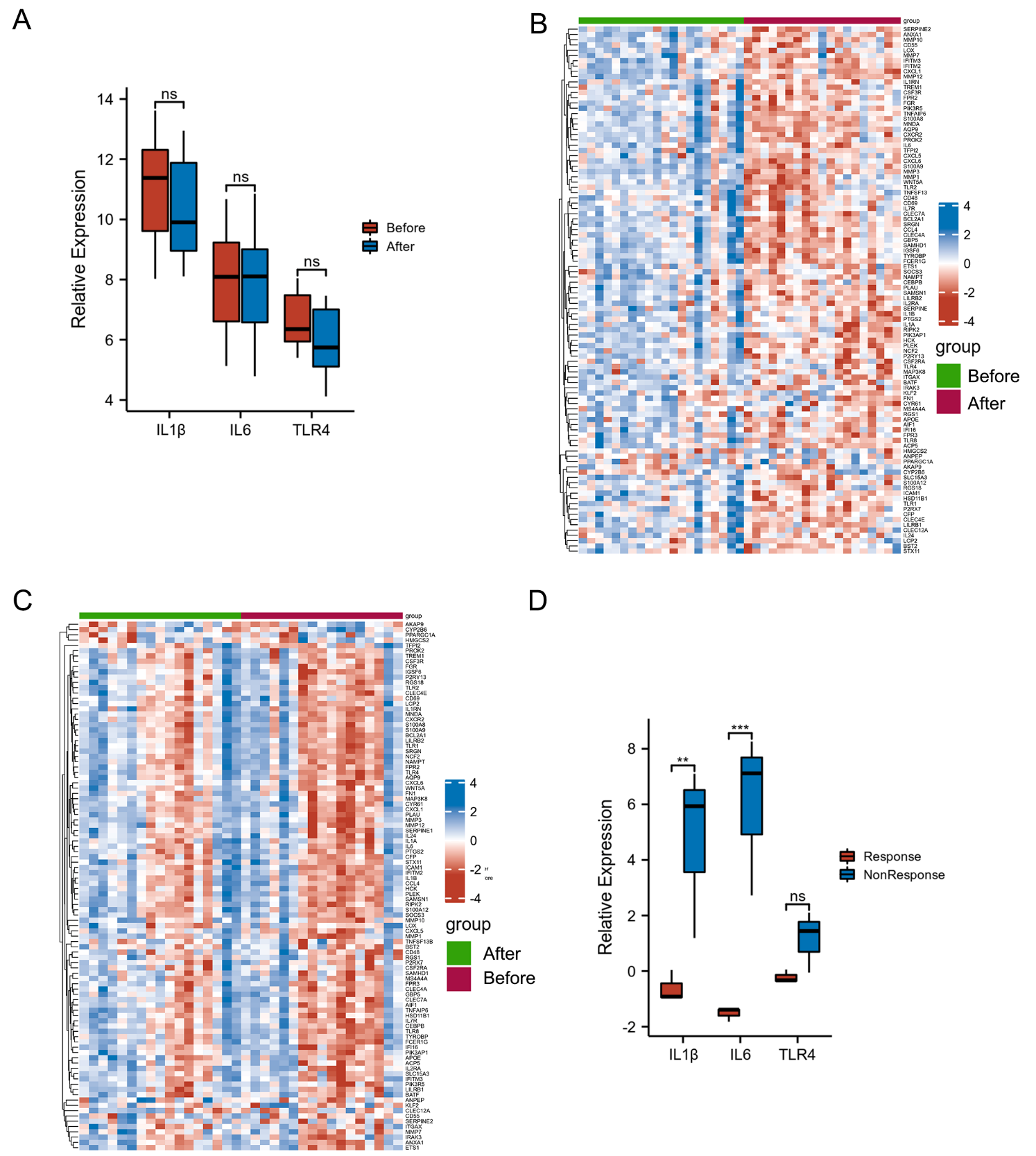


**Supplementary Figure S5.** (A) The relative expression of IL1β, IL6, and TLR4 before and after treatment in nonresponse group. (B) The heatmap of top 100 nodes before and after treatment in response group. (C) The heatmap of top 100 nodes before and after treatment in nonresponse group. (D) The relative expression of IL1β, IL6, and TLR4 before and after treatment in GSE111761. (**p < 0.01, ***p < 0.001, ns, not significant)
